# Pulmonary and pleural lymphatic endothelial cells from pediatric, but not adult, patients with Gorham-Stout disease and generalized lymphatic anomaly, show a high proliferation rate

**DOI:** 10.1186/s13023-016-0449-4

**Published:** 2016-05-18

**Authors:** Michiko Mori, Michael Dictor, Nicholas Brodszki, Juan Carlos López-Gutiérrez, María Beato, Jonas S. Erjefält, Erik A. Eklund

**Affiliations:** Department of Experimental Medical Sciences, Unit of Airway Inflammation, Lund University, Lund, Sweden; Department of Clinical Sciences, Section for Pathology, Lund University, Lund, Sweden; Department of Clinical Sciences, Section for Pediatrics, Lund University, Lund, Sweden; Vascular Anomalies Center, La Paz Children’s Hospital, Madrid, Spain; Department of Pathology, La Paz Children’s Hospital, Madrid, Spain

**Keywords:** Gorham-Stout disease, Generalized lymphatic anomaly, Lymphatic malformation, Chylothorax, VEGF-C

## Abstract

**Background:**

Gorham-Stout disease (OMIM 123880) and generalized lymphatic anomaly are two rare disorders of lymphendothelial growth in which thoracic involvement with chylothorax is a feared complication. Currently it is believed that both disorders are prenatal malformations that progress slowly after birth. Several pharmaceuticals with antiproliferative properties, including interferon-α-2b, rapamycin and propranolol, have however been shown to affect the disease course in some patients. Deeper knowledge of the growth characteristics of these malformations are therefore needed to guide the clinical approach.

**Methods:**

Lymphatic vessels in lung and pleural tissue from both children and adult patients with generalized lymphatic anomaly or Gorham-Stout disease were studied using an immunohistochemical approach, targeting lymphendothelial markers (D2-40/Prox-1) and a proliferation marker (Ki-67).

**Results:**

We found significant proliferation and growth in these lesions in pediatric patients but not in adults. Furthermore, the data may suggest that the disease process is at least partly reversible.

**Conclusions:**

These malformations of the lymphatic system proliferate at a significant rate long after birth, which could suggest that the clinical approach for children should be different from adults.

**Electronic supplementary material:**

The online version of this article (doi:10.1186/s13023-016-0449-4) contains supplementary material, which is available to authorized users.

## Background

Lymphatic malformations (LM) constitute a broad group of disorders, ranging from single lesions to wide spread disease [1]. Systemic conditions include generalized lymphatic anomaly (GLA), Gorham-Stout disease (GSD; OMIM 123880) and Kaposiform lymphangiomatosis (KLA), all considered to be very rare entities [2–4]. The classification of these conditions is challenging, the literature uses descriptive and inconsistent terminology, and few papers describe more than a small number of patients. GSD is an LM characterized by progressive osteolysis, whereas the related condition, GLA, also may have skeletal involvement, however without progressive loss of cortical bone [5]. Both conditions are thought to be congenital and the general belief is that the lymphatic endothelial cells (LECs) of these LMs do not dived rapidly after birth, although their clinical course is progressive in nature [6]. Symptoms may occur from infancy into adulthood. In both GLA and GSD a feared complication of intrathoracic involvement is effusion of chyle into the pleural cavity (chylothorax). This condition is often difficult to manage [7] and is associated with high morbidity and mortality. In the literature there are many suggested therapies ranging from drainage, pleurodesis and radiotherapy [6] to pharmaceutical drugs such as interferon-α-2b [8], propranolol [9], rapamycin [2], low-molecular weight heparin [7] and anti-VEGF antibodies [10]. Even though the endothelial cells of these lesions are thought to have a low rate of division, many of the pharmaceuticals used (and reported to be at least partly efficient) strongly affect cell proliferation. We therefore set out to study whether LECs in patients with either GLA or GSD really were semi-quiescent and if not, whether there was an age difference. We also studied biopsy material before and after treatment in a young girl with GLA to determine whether these processes are potentially reversible.

## Methods

### Study cohort

There are no large collections of fixed tissue from patients with GSD or GLA, which makes statistical analysis of immunohistochemical results challenging. We set out to create a biobank of formalin-fixed material in these conditions, including tissue from the skeleton, bone marrow, spleen and soft tissues (e.g., skin, fat and connective tissue). Material was retrieved from pathology laboratories in Sweden, Spain, Italy and the biobank NDRI (National Disease Research Interchange) in Philadelphia, USA. The tissue origin and patient data are presented in Table 1. In the collection there is also material from peripheral parts of lungs or pleurae from 8 patients, including 5 children (6 months-16 years of age) and 3 adults (>23 years of age), all of whom had developed chylothorax. Clinical details are listed in Table 2. In total, material from 23 GSD and GLA patients was collected and is now available for collaborative studies. As controls for this study, tissue blocks of normal lung and pleura were obtained from the Dept. of Pathology (Lund, Sweden; 2 pediatric and 3 adult patients), Sahlgrenska University Hospital (Gothenburg, Sweden; 2 adult patients), and the University Hospital of Umeå (Umeå, Sweden; 1 pediatric patient). Two pediatric controls died from complications not related to the lungs (acute cardiac arrest) and one was investigated for isolated pericardial effusion. Four adult patients succumbed from causes not related to the pulmonary system and their lungs were taken for donation, whereas one died from pneumothorax.

**Table 1:** Collection of non-pulmonary specimens from patients with Gorham-Stout disease or generalized lymphatic anomaly

Subject	Original diagnosis (GSD ^a^, GLA ^b^)	Tissue	Gender (M/F)	Age (yrs.)^c^
1	GSD	Connective tissue	M	48
2	GSD	Connective tissue	F	6
3	GSD	Leg	F	56
4	GSD	Bone marrow	F	34
5	GSD	Frontal bone, spleen	F	5
6	GSD	Cervical bone	M	5
7	GSD	Adipose tissue	M	unknown
8	GSD	Bone, connective tissue	M	16
9	GSD	Skin	F	8
10	GSD	Soft tissue	unknown	unknown
11	GSD	Skin, soft tissue	M	unknown
12	GSD	Skin	M	15
13	GSD	Bone	F	14
14	GSD	Bone, soft tissue	F	10
15	GSD	Skin	M	4
16	GLA	Bone marrow	F	4
17	GLA	Bone, crista	M	3
18	GSD	Bone, soft tissue	M	0.5

^a^Gorham-Stout disease

^b^Generalized lymphatic anomaly

^c^Age at diagnosis

**Table 2:** Collection of lung and pleural specimens from patients with Gorham-Stout disease or generalized lymphatic anomaly, and controls

Subject	GSD ^a^/GLA ^b^/Control	Gender (M/F)	Age (yrs.) ^c^
1	GLA	M	3
2	GLA	F	54
3	GSD	F	Infant
4	GLA	F	4
5	GLA	M	3
6	GLA	F	35
7	GLA	F	57
8	GSD	M	0.5
9	Control	M	0.5
10	Control	M	23
11	Control	M	25
12	Control	M	36
13	Control	F	8
14	Control	M	16
15	Control	M	65
16	Control	F	63

^a^ Gorham-Stout disease

^b^ Generalized lymphatic anomaly

^c^ Age at diagnosis

### Immunohistochemistry (IHC)

Tissue samples were fixed in 10 % formalin, dehydrated and embedded in paraffin. In total, 46 tissue blocks were included in the study; 32 tissue blocks from patients with GSD or GLA and 14 tissue blocks from controls. Paraffin sections of three-micron-thickness were heated to 60 °C for 20 min and then subjected to simultaneous dewaxing and heat-induced antigen retrieval in high pH (EnVision FLEX Target Retrieval Solution, K8010, Dako, Glostrup, Denmark) using a Dako Pre-Treatment Module. To avoid staining variations between slides, all immunohistochemical stainings were carried out in an automated slide staining robot (Autostainer Plus, DakoCytomation, Glostrup, Denmark) and each IHC run contained sections from both study groups. Adjacent tissue sections were first treated with 0.3 % hydrogen peroxide for 10 min to block endogenous peroxidase activity and then incubated for 1 h with antibodies directed against Prox1 (1:600, rabbit polyclonal, ab38692, Abcam, Cambridge, UK) or Ki-67 (1:150, mouse monoclonal, M7240, Dako, Glostrup, Denmark). Next, sections were treated with Polymer/HRP-linked secondary antibodies (K8010, Dako) for 30 min and immunoreactivity was visualized with 3,3′-diaminobenzidine (DAB) chromogen (brown-colored product, K8010, Dako). An additional blocking step with Denaturating Solution kit (DNS001L, Biocare Medical, Concord, CA, USA) for 5 min was performed to prevent additional binding to the first primary antibody [11, 12]. Sections were then incubated with mouse anti-human D2-40 antibodies (1:300, CM266, Biocare Medical) and immunoreactivity was visualized with Polymer/HRP-linked secondary antibodies (K8010, Dako) and Vina Green Chromogen kit (green-colored reaction, BRR807AS, Biocare Medical). Finally, sections were counterstained with Mayer’s hematoxylin, air-dried and mounted with Pertex.

### Quantitative assessments of lymphatic vessels

Whole sections were digitalized using a slide-scanning robot (ScanScope Slide Scanner, Aperio Technologies, Vista, CA, USA). Morphometric and immunohistochemical measurements were performed on the generated digital images using Aperio ImageScope Image Analysis Software (V.10.0, Aperio Technologies) [11, 12].

Total D2-40 immunoreactivity in whole sections was assessed by manually delineating the perimeter of the whole tissue and calculating the percentage of the total tissue area occupied by D2-40-staining (i.e. the number of green-colored positive pixels related to the total number of tissue pixels) using Aperio Positive Pixel Count Algorithm v.9 (ImageScope, Aperio Technologies) (see Additional file 1). Pixels corresponding to Prox1 immunoreactivity (brown color) were automatically excluded in the analysis.

The numbers of Prox1^+^/D2-40^+^ lymphatic vessels in each section were counted and data were normalized to the tissue area, which was assessed by counting the number of pixels corresponding to the tissue (i.e., excluding air/luminal spaces). In lung sections, all bronchioles (absence of cartilage and diameter <2 mm) and bronchiole-associated arteries, as well as multiple solitary blood vessels (at a distance from bronchioles) and large areas of alveolar tissue were analyzed on each section. In pleural sections, four randomly selected areas (about 3.6 mm^2^ each) were analyzed on each section. In the same regions the numbers of lymphatic vessels showing at least one Ki-67^+^ lymphatic endothelial cell nucleus were counted to reveal the percentage of lymphatic vessels in active proliferation.

### Statistics

Statistical analysis was performed using GraphPad Prism V.6.0 (GraphPad software, San Diego, CA, USA). Mann-Whitney rank sum nonparametric test was used for comparison between the two study groups. Values are given as median (range), unless otherwise stated.

## Results

### Lymphatic volume

**Fig. 1:**
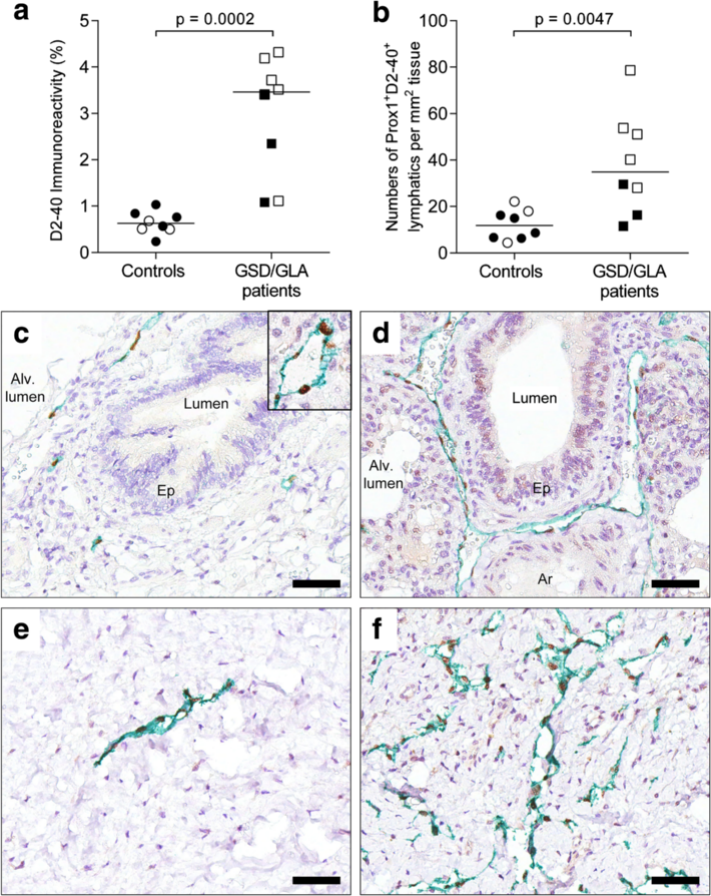
Lymphatic volume in patients with Gorham-Stout disease (GSD) and generalized lymphatic anomaly (GLA). **a** Quantification of the total tissue immunoreactivity for D2-40^+^ lymphatic endothelial cells in lung and pleural tissue. **b** Numbers of Prox1^+^/D2-40^+^ lymphatic vessels normalized to the lung/pleural tissue area. Statistical analysis was performed using Mann-Whitney rank sum test. Horizontal lines indicate median values. Open symbols: children (6 months-16 years of age). Black symbols: adults (>23 years of age). **c**-**f** Immunohistochemical staining for Prox1 (*brown*-*colored* nuclei, see inlet of Fig. 1c) and D2-40 (in *green*) in controls (*left panel*) and patients with GSD/GLA (*right panel*). Lymphatics with long vessel perimeter are exemplified in (**d**) and (**f**). Representative photomicrographs of histological sections of lung tissue (in **c**-**d**) and pleural tissue (in **e**-**f**). Cell nuclei were counterstained with Mayer’s hematoxylin (*blue*). Scale bars: (**c**-**f**) 50 μm

First, we set out to study the area of tissue that was occupied by lymphatic vessels using the LEC marker D2-40. The area was defined as the D2-40 positive area/total tissue area (excluding air and luminal spaces) (Fig. 1a, Additional file 1). In control samples from both lung tissue and pleurae the average area covered by lymphatic vessels was 0.6 % (Fig. 1a). In patients with GLA or GSD this area was considerably larger, with an average of 3.5 %, *p* = 0.0002 (Fig. 1a). Further, the number of Prox1^+^/D2-40^+^ vessels per mm^2^ tissue also was clearly increased in patients compared to controls (38 versus 12; *p* = 0.0047) (Fig. 1b). Interestingly, there was a clear trend towards fewer lymphatic vessels per mm^2^ in adult tissue than in tissue from children. The lymphatic vessel perimeter was often larger in patients compared to controls. The typical immunohistochemical appearance of the lymphatic vessels is shown in Fig. 1c-f.

### Proliferation rate

**Fig. 2:**
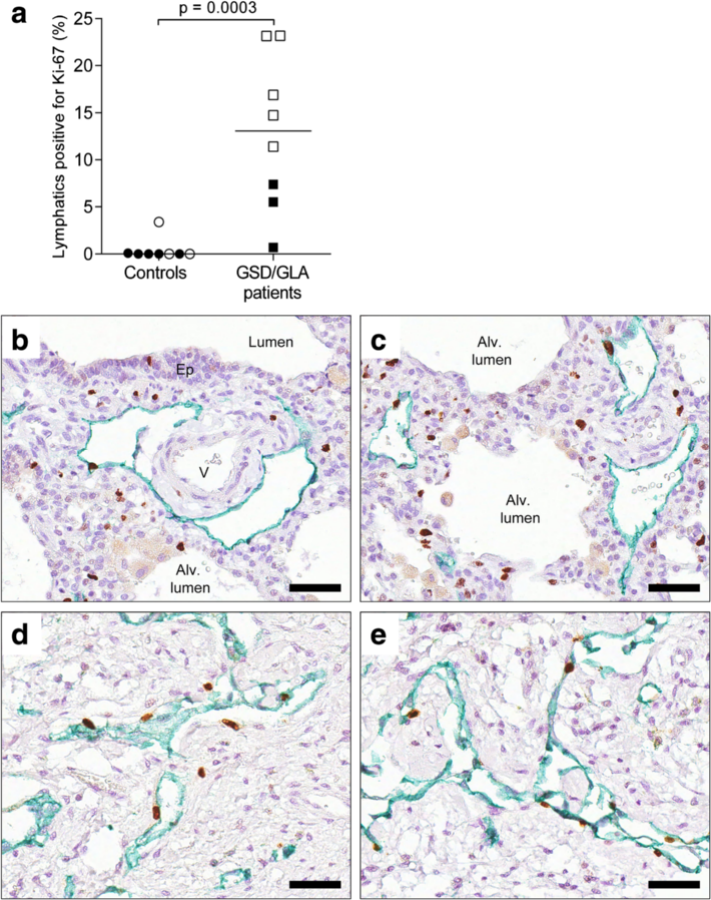
Lymphatic proliferation rate in patients with Gorham-Stout disease (GSD) and generalized lymphatic anomaly (GLA). **a** Quantification of lymphatic vessels with actively proliferating lymphatic endothelial cells in lung and pleural tissue. Statistical analysis was performed using Mann-Whitney rank sum test. Horizontal lines indicate median value. Open symbols: children (6 months-16 years of age). Black symbols: adults (>23 years of age). **b**-**e** Immunohistochemical staining for Ki67 (*brown*-*colored* nuclei) and D2-40 (in *green*) in patients with GSD/GLA. Representative photomicrographs of histological sections of lung tissue (**b**-**c**) and pleural tissue (**d**-**e**). Cell nuclei were counterstained with Mayer’s hematoxylin (*blue*). Scale bars: (**b**-**e**) 50 μm

Proliferation of LECs was assessed using double staining for D2-40 and the proliferation marker Ki-67. In the control group, except for one sample analyzed, there was no significant proliferation at all. In GLA/GSD patients, however, 12.5 % of the lymphatic vessels on average contained proliferating cells (Fig. 2). There was a significant difference (*p* = 0.036) between samples from adult and pediatric patients, where the adult patients on average showed proliferating cells in 5 % of the lymphatic vessels whereas the pediatric population on average had 17.5 %.

### Effect of antiproliferative treatment

**Fig. 3:**
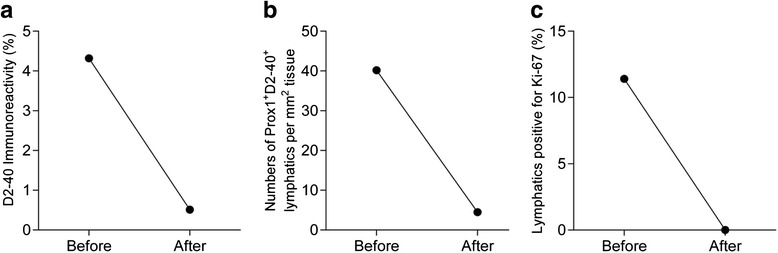
Antiproliferative treatment effects on lung and pleural lymphatic vessels in a 4-year-old with generalized lymphatic anomaly. **a** Quantification of the total tissue immunoreactivity for D2-40^+^ lymphatic endothelial cells before and after antiproliferative treatment. **b** Numbers of Prox1^+^D2-40^+^ lymphatic vessels normalized to the tissue area. **c** Quantification of lymphatic vessels with actively proliferating lymphatic endothelial cells

As the aberrant lymphatics in GSD/GLA are thought to be malformations with a low rate of proliferation, we studied this feature in lung/pleural tissue before and after treatment in a young girl suffering from GLA, whose clinical history has been published (Brodszki et al., 2011, case 2 [7]). In short, a female patient was diagnosed at four years of age with bilateral chylothorax after persistent back pain. The initial CT-scan revealed several fractured ribs, sternum fracture and diffuse osteolytic changes in the humerus, femur, pelvis, sacrum and multiple vertebrae. Cystic changes were noted in the spleen as well. The diagnosis of GSD was made on the basis of the clinical symptoms, radiological findings and histology. In retrospect, the correct diagnosis was GLA following the criteria by Lala et al. [5] as the bone lesions were not progressively osteolytic. The patient was treated with radiotherapy, octreotide, interferon-α − 2b/pegylated interferon and tafoxiparin as described in the initial publication [7]. Her chylothorax resolved permanently but the osteolytic changes never remitted and she became paraplegic 3.5 years later after collapse of the thoracic spine (at Th10). The treatment was broadened to include daily rapamycin, propranolol and triweekly intravenous infusion of pamidronate. She succumbed to sepsis, which was deemed unrelated to the GLA. At a restricted necropsy, tissue from the lung/pleurae was harvested and analyzed for presence of lymphatic malformations. This was then compared to the tissue submitted at the time of diagnosis, which showed that on average 4 % was made up of lymphatic (D2-40^+^) cells, whereas only 0.5 % of the tissue was D2-40^+^ at the time of death (Fig. 3a). Further, the number of lymphatic vessels per mm^2^ tissue, decreased from 40 to 5 between diagnosis and death (Fig. 3b) and the percentage of actively proliferating lymphatic vessels went from 11 % to null (Fig. 3c). These data indicate thus that although the number of vessels and their proliferative activity were elevated at diagnosis, both parameters were reversible at this stage.

## Discussion

In the latest classification from the International Society for the Study of Vascular Anomalies (ISSVA) [1], GLA and GSD are classified as two different disorders, though they share many features. Furthermore, the related and seemingly even more aggressive LM, KLA, may share features of these two other conditions [3, 4]. The diagnostic differences between GLA and GSD include the ‘most common skeletal location’ and ‘radiographic appearance of the skeletal lesions’ [5]. The skeletal disease course appears more aggressive in GSD, while the diagnosis of GLA may allow for await-and-see approach rather than intervention [13]. However, pleuropulmonary involvement, when present, seems to be indistinguishable in the two conditions, which offered us a rationale for combining the material from GLA and GSD patients to augment the study cohort. None of the studied patients presented with foci of spindled LECs and KLA was therefore ruled out [4]. There is no standardized treatment for these often-fatal conditions, and several approaches have been used over the years. These include pharmacological substances such as interferon-α-2b [8], propranolol [9, 14], rapamycin [2], and bevacizumab [10], but also local radiotherapy [15], sclerosing therapy [16] and ligation of the thoracic duct (in chylothorax) [17]. Recently, a combination of sunitinib and taxol was also suggested [13]. Despite the fact that most of these therapies exert their potentially beneficial effects as anti-proliferation agents, the lymphatic lesions in GLA and GSD are considered to be slowly dividing malformations rather than highly proliferating tumor-like structures. To study whether this is true in all age groups and whether the process is reversible we first collected specimens from different organs in patients of various ages, in order to create a tissue biobank from these rare patients for use by the scientific community. At present, we have formalin-fixed paraffin-embedded tissue from 23 patients as described in Tables 1 and 2. In our biobank we then identified material from eight patients with pleuropulmonary involvement and compared them to pulmonologically healthy age-matched individuals. The total tissue area covered by lymphatic vessels was increased four-fold over normal tissue (3.5 % compared to 0.8 %), a relation apparently not previously quantified. This was accompanied by a significantly increased number of lymphatic vessels per mm^2^ of tissue in the patients, indicating the strong impact of these disorders on lymphangiogenesis. These findings, together with the observation that the lymphatic vessel perimeter appeared to be larger in patients, may indicate that the increase in total volume is not only caused by the LMs containing more vascular structures but also wider vessels. Using double staining for D2-40 and Ki-67, we could show that the number of actively proliferating lymphatic vessels in tissue from pediatric patients was clearly higher than in the adult population (18 % vs 5 %), whereas there were virtually no Ki-67-positive LECs in the controls. This indicates that there is an intensive expansion of the pleuro-pulmonary LMs during the first decades of life. Drugs affecting this process are thus more likely to be effective at a younger age.

The factors driving the proliferation of lymphatic vessels in patients with GSD or GLA are not known but may include known lymphangiogenic cytokines such as vascular endothelial growth factor (VEGF)-A, VEGF-C, VEGF-D and platelet-derived growth factor (PDGF)-BB [18]. In line with this we previously reported increased serum levels of VEGF-A in two pediatric patients with GLA and increased levels of VEGF-C in one patient [7]. Another study showed that lymphatic vessels in a GSD patient expressed both VEGFR3 and PDGFR-β, in addition to receptors for VEGF-C/D and PDGF-BB [19]. An interesting animal model of the related human disorder pulmonary lymphangiectasia [20], using perinatal overexpression of VEGF-C, implies the involvement of both VEGFR2 and VEGFR3 in aberrant pulmonary lympangiogenesis. Whether this model can be used to mimic the pathogenesis of GLA/GSD is not known and future studies need to define the mechanisms causing the lymphatic proliferation in patients with these conditions.

In a deceased patient, we could show that the LM-forming process was reversible, as the infiltrating LMs at diagnosis were largely absent at the time of death. Whether this was caused by the GLA targeted treatment or non-related factors associated with the death of the patient is however impossible to determine postmortem in a single case.

In conclusion, we stress the possible importance of an early diagnosis and treatment, as opposed to the ‘await and see’ philosophy suggested by Rössler et al. [13], since waiting may render the disease less treatable. Indeed, in our clinical experience, many of the pharmacological approaches suggested in the literature have been largely ineffective in adult patients with chylothorax. Our data also may suggest that children and adults with GLA/GSD and chylothorax should be treated differently. The number of patients in this study is however limited and would ideally be expanded to further strengthen this conclusion.

## Conclusions

In this paper we describe the creation of a tissue biobank with material from in total 23 patients with GLA or GSD, which is open for collaborative ventures to study these rare entities. We focused on LMs in the pleuropulmonary system of GLA and GSD patients and show that there is significant LM proliferation during childhood, but not in adults, which may have implications for the choice of therapy.

## Ethics approval and consent to participate

The research was approved after vetting by The Central Ethical Review Board at Lund University, Lund, Sweden (2013/58). Parental or patient consent was waivered in cases where the consenter was unavailable. All other patients or patient guardians gave informed consent to the study.

## Consent for publication

Parental or patient consent was waivered in cases where the consenter was unavailable. All other patients or patient guardians gave informed consent to the publication of this study.

## Availability of data and supporting materials

Not applicable.

## Additional file

Additional file 1: Assessment of total D2-40 immunoreactivity in whole tissue sections. (*A*) Double staining immunohistochemistry for the lymphatic endothelial nuclei marker Prox1 (brown nuclei) and the lymphendothelial cell surface marker D2-40 (green). Cell nuclei were counterstained with Mayer’s hematoxylin (blue). (*B*) The corresponding computerized color segmentation using Aperio Positive Pixel Count Algorithm v.9 on the same image as in *A*. The tissue structures and immunoreactivity have been replaced with pseudo colors: nuclei, background tissue and Prox1^+^ immunoreactivity are shown in blue, and D2-40^+^ immunoreactivity in red, orange and yellow (corresponding to the descending intensity of the green-colored immunopositive pixels). (TIF 3949 kb)

## Abbreviations

GLA: generalized lymphatic anomaly
GSD: Gorham-Stout disease
IHC: immunohistochemistry
LEC: lymphatic endothelial cell
LM: lymphatic malformation
VEGF: vascular endothelial growth factor
