# Supplementary figures and images for: Pulmonary and pleural lymphatic endothelial cells from pediatric, but not adult, patients with Gorham-Stout disease and generalized lymphatic anomaly, show a high proliferation rate
Source: Orphanet J Rare Dis. 2016 May 18;11:67. doi: 10.1186/s13023-016-0449-4 (PMC4870727; doi:10.1186/s13023-016-0449-4)

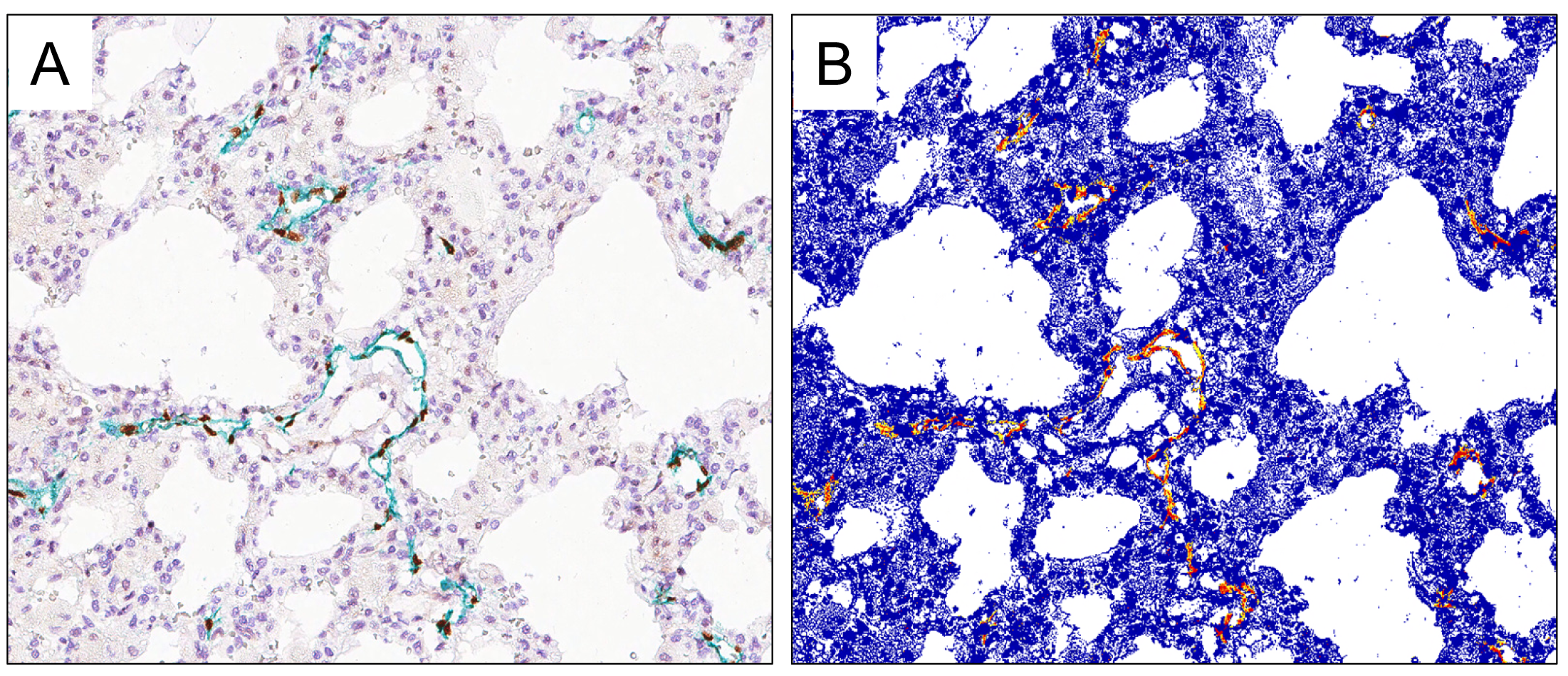

Supplement: Additional file 1: — Assessment of total D2-40 immunoreactivity in whole tissue sections. (A) Double staining immunohistochemistry for the lymphatic endothelial nuclei marker Prox1 (brown nuclei) and the lymphendothelial cell surface marker D2-40 (green). Cell nuclei were counterstained with Mayer’s hematoxylin (blue). (B) The corresponding computerized color segmentation using Aperio Positive Pixel Count Algorithm v.9 on the same image as in A. The tissue structures and immunoreactivity have been replaced with pseudo colors: nuclei, background tissue and Prox1+ immunoreactivity are shown in blue, and D2-40+ immunoreactivity in red, orange and yellow (corresponding to the descending intensity of the green-colored immunopositive pixels). (TIF 3949 kb) [file 13023_2016_449_MOESM1_ESM.tif]
